# Alterations of liver enzymes and lipid profile in response to exhaustive eccentric exercise: vitamin D supplementation trial in overweight females with non-alcoholic fatty liver disease

**DOI:** 10.1186/s12876-022-02457-w

**Published:** 2022-08-05

**Authors:** Zahra Rahimpour, Rastegar Hoseini, Nasser Behpour

**Affiliations:** Department of Exercise Physiology, Faculty of Sport Sciences, Razi University, Kermanshah, Iran

**Keywords:** NAFLD, Exercise, Vitamin D, Liver enzymes, Lipid profile

## Abstract

**Background:**

Eccentric exhaustive exercise (EEE) training has been known as a promising training modality to enhance performance and stimulate adaptation in healthy individuals or patients that might also cause abnormal liver enzymes and lipid profiles. Vitamin D (Vit D) supplementation is believed to improve the condition of Non-Alcoholic Fatty Liver Disease (NAFLD) patients. However, there is limited evidence on the effect of Vit D supplementation on the EEE-induced alterations. This study aimed to investigate the effect of short-term supplementation of Vit D on the liver enzymes and lipid profile alterations following EEE in overweight women with NAFLD.

**Methods:**

In this clinical trial, 22 overweight women with NAFLD were randomly divided into experimental and control (n = 11 in each). The experimental group consumed 2000 IU of Vit D per day for six weeks; the control group consumed a lactose placebo daily with the same color, shape, and warmth percentage. Two treadmill EEE sessions were performed before and after the six-week Vit D supplementation. Blood was taken from the antecubital vein to measure the liver enzymes, lipid profile, and Vit D at four stages: Pre 1(before the first EEE session), Post 1(after the first EEE session), Pre 2 (before the second EEE session), and Post 2 (after the second EEE session).

**Results:**

The results indicate that Vit D supplementation significantly reduced Bodyweight (BW; *P* = 0.047), Body Mass Index (BMI; *P* = 0.044), Body Fat Percentage (BFP; *P* = 0.001), and Waist Hip Ratio (WHR; *P* = 0.001) in the experimental group. Additionally, the results showed increased liver enzymes (ALT, AST, and GGT) and lipid profile (TC, TG, and LDL) following EEE. While the HDL levels decreased significantly after EEE. Compared with control, the results of the independent t-test showed significantly lower ALT (*P* = 0.001; *P* = 0.001), AST (*P* = 0.001; *P* = 0.001), and GGT (*P* = 0.001; *P* = 0.001); while significantly higher Vit D (*P* = 0.001, *P* = 0.001) in the experimental in both Pre 2 and Post 2; receptively. Also, significantly lower TC (*P* = 0.001; *P* = 0.001), TG (*P* = 0.048; *P* = 0.001), and LDL (*P* = 0.001; *P* = 0.001); while significantly higher HDL (*P* = 0.001, *P* = 0.001) were observed in the experimental group compared to the control in both Pre 2 and Post 2; receptively.

**Conclusions:**

Vit D supplementation reduces the liver enzymes and improves lipid profile alterations following EEE in overweight women with NAFLD. Thus, Vit D supplementation can be considered a functional supplement to improve the EEE-induced alteration.

*Trial registration*: The trial was in the Iranian Clinical Trial Registration Center under the (IRCT20201130049538N1) on 05/07/2021.

## Background

Non-alcoholic fatty liver disease (NAFLD) is a prevalent liver condition that affects 3–25% of the general population and is thought to be the leading cause of liver cancer and cirrhosis [1]. Visceral fat accumulation accompanied by overweight and obesity is a strong risk factor for NAFLD [2]. Although no definitive treatment has been found for NAFLD, a combination of exercise and diet seems to be non-pharmacological approaches to prevent and control the condition [3, 4].

Different types of exercises create various responses and adaptations [5, 6]; Eccentric Exhaustive Exercise (EEE) has recently been introduced as a new exercise therapy inducing weight loss in a shorter period. Despite the positive effect of long-term EEE on weight loss and physiological adaptation, single bouts of EEE induce cell damage [7] that creates a transient increase in liver enzymes and lipid profile levels [8]. According to studies, EEE bouts reduce hepatic blood flow acutely (regulated by endothelin-1) and increase hepatocyte mitochondrial swelling, which leads to liver damage due to the rupture of the mitochondrial membrane and the release of pro-apoptotic proteins (e.g., cytochrome c) into the cell, resulting in liver damage [9, 10]. Additionally, EEE-induced cell damage increases liver enzyme releasement into the bloodstream, causing structural protein changes, decreased performance, inflammation, muscle fatigue, and inflammation, also known as a clinical indicator of cardiac and hepatic damage [11, 12].

Additionally, among the dietary supplements, vitamin D (Vit D) plays a significant role in preventing several diseases including NAFLD [13]. According to Vatandost et al. (2018) meta-analysis, the prevalence of Vit D deficiency in the total population was 56% [14]. Vit D modulates inflammation, reduces parathyroid hormone levels, increases insulin sensitivity, and improves lipid profile [15]. Vit D also prevents fat deposition in the liver by enhancing fat oxidation, inhibiting lipogenesis, and regulating the circulation of free fatty acids [9].

Previous studies showed that long-term Vit D supplementation has an anti-fibrotic and anti-inflammatory role that improves lipid profile and liver enzymes in NAFLD patients [10, 16]. However, few studies have investigated the effect of short-term Vit D supplementation on EEE-induced lipid profile and liver enzyme alterations in NAFLD patients. Therefore, in this study, we conducted a randomized controlled trial (RCT) to investigate the effect of short-term Vit D supplementation on the liver enzymes and lipid profile responses to EEE in overweight women with NAFLD.

## Methods

### Ethical approval

The Ethics Committee of the Kermanshah Razi University approved this trial (IR.RAZI.REC.1399.079). It was also registered in the Iranian Clinical Trial Registration Center under the IRCT20201130049538N1 on 05/07/2021. All participants signed a written informed consent form that stated their willingness to participate voluntarily and the possibility of withdrawing from the study.

### Study design and participants

**Fig. 1:**
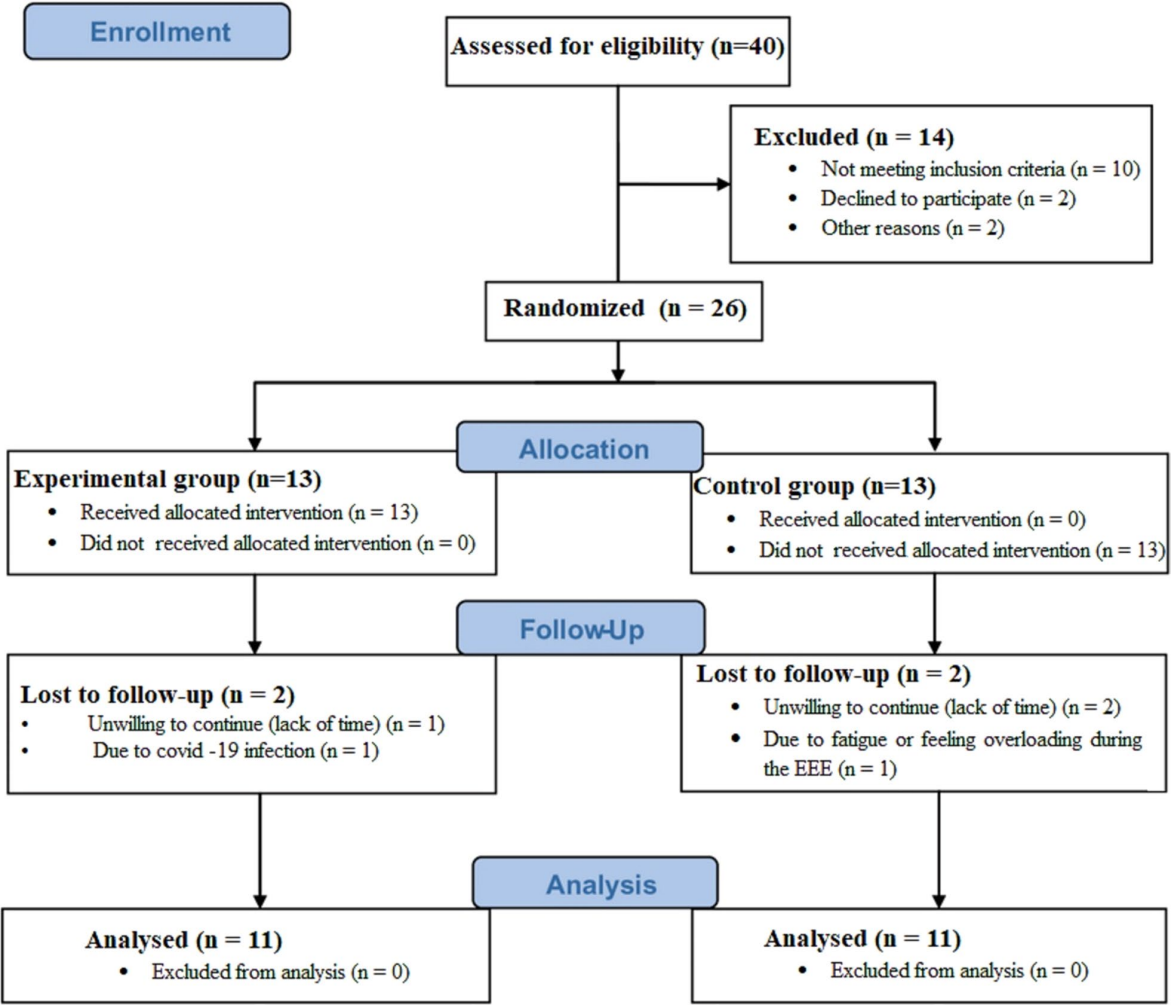
Consort flow diagram for the study

This study is a single-blinded quasi-experimental with Pre-test and Post-test design, with an experimental and a control group. Forty overweight women (aged 20–40 years, Kurd ethnicity, skin phototypes II–IV, manly students or graduates, with low sun exposure owing to clothing and lifestyle, with a regular sleeping pattern) volunteered to participate in the study after finding out about the participation announcement over social media. Inclusion criteria included being diagnosed with NAFLD using ultrasound imaging and having Body Mass Index (BMI) between 25.0 and 29.9 kg/m^2^. Exclusion criteria were smoking, history of heart or kidney disease, taking lipid-lowering drugs, being infected with COVID-19, Vit D supplementation and regular exercise six months before the start of the study, fundamental diet changes, and failure to follow the study protocol. Twenty-six individuals who met the inclusion criteria were then selected as subjects divided into two groups of 13; the experimental group and the control. Then, the subjects completed the written informed consent and related questionnaires. It should be noted that two individuals in each group refused to continue the study. The consort flow diagram for the study is shown in Fig. 1. The enrollment workflow to the program and exclusion criteria is provided in Fig. 1.

### Body composition

Height was calculated using a wall tape measure with a minimum of 0.1 cm. Also, Bodyweight, BMI, Body Fat Percentage (BFP), and Waist–Hip Ratio (WHR) were assessed using a bioelectric impedance device (In Body Korea).

### EEE session protocol

Two sessions (one session before supplementation and one session after) of EEE training were performed on the treadmill with a negative slope. After a 5-min warm-up on the treadmill with a zero slope and a speed of 3 km/h, the test began at a speed and slope of 4 km/h and − 2°, with − 2° added to the slope and 1 km/h added to the speed every three minutes. The test continued until exhaustion. Finally, a 5-min cool-down was performed at a speed and slope of 3 km/h and zero. The polar heart rate monitor and Borg scale were used to control the exercise intensity [7]. All training sessions were performed between 8 and 11 in the morning, at the fasting state, and at a temperature of 20–25 °C in the sports sciences faculty laboratory, Razi University, Kermanshah.

### Vit D supplementation

**Fig. 2:**
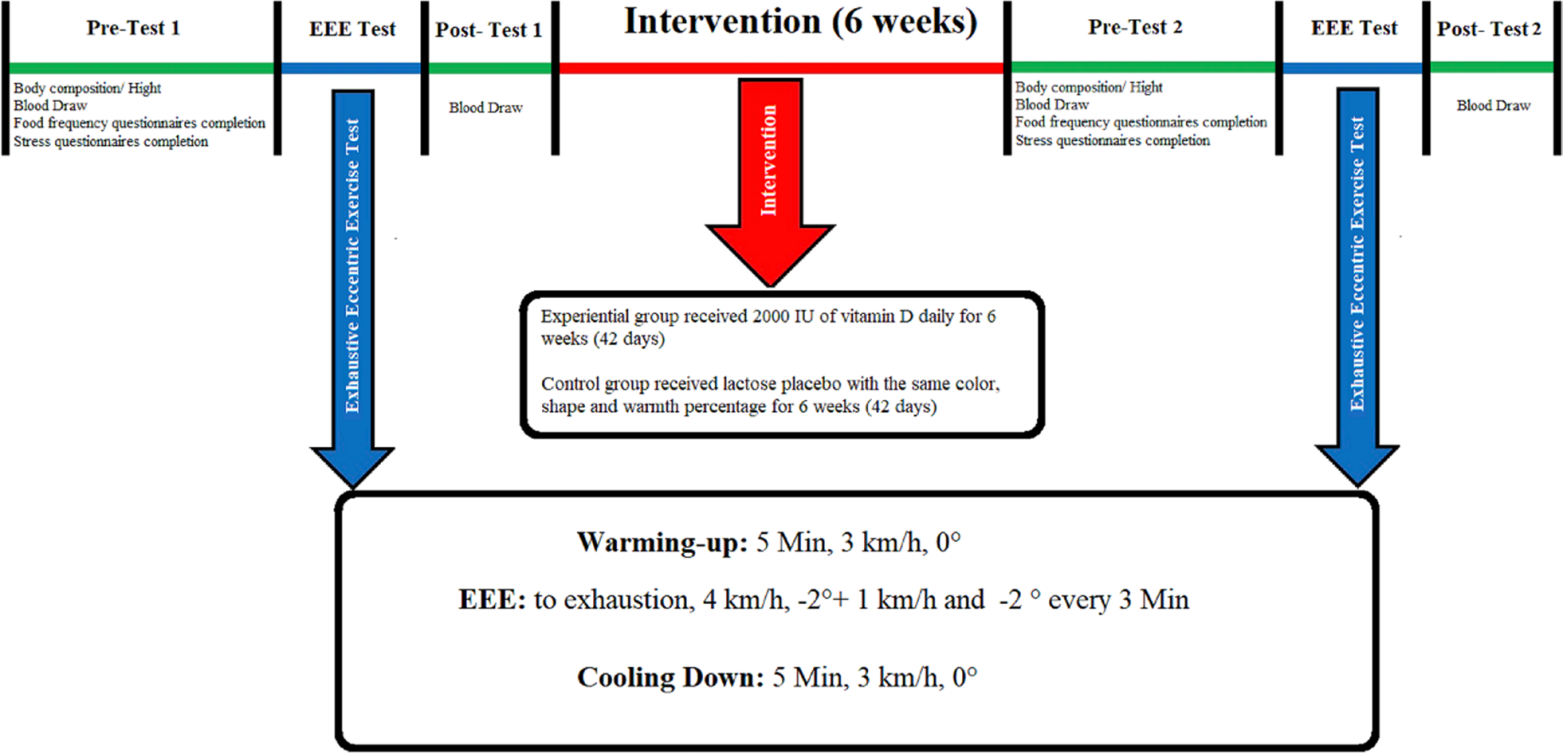
Overview of the study design

In this study, the Vit D supplement group consumed 2000 IU of Vit D per day (Zahravi Pharmaceutical Company) for six weeks. The control group also consumed a daily dose of lactose placebo with the same shape, color, and warmth percentage as the Vit D [7]. The subjects completed the 3-day food frequency questionnaire before the intervention. Subjects were asked to consume the same food and the same amount of calories one day before the blood sampling, Pre and Post-tests. The subjects' diet consisted of 55% carbohydrates, 30% fat, and 15% protein. Figure 2 shows the overview of the study design.

### Measurement of blood samples

Blood samples were taken at 4-time points; before the first training protocol (Pre1), immediately after the first training protocol (Post 1), before the second training protocol (Pre2), and immediately after the second training protocol (Post 2). Due to the possible effects of the menstrual cycle, all blood samplings were made out during the menstruation phase. Blood samples were collected in heparinized tubes and stored at -80˚C for further lipid profile and liver enzyme measurements. To measure the liver enzymes (Alanine Aminotransferase (ALT; Intra- and inter-assay coefficients of variation of 5.1% and 3.8%), Aspartate Aminotransferase (AST; Intra- and inter-assay coefficients of variation of 6.5% and 3.8%), and Gamma-Glutamyl Transferase (GGT; Intra- and inter-assay coefficients of variation of 5.3% and 3.0%)) the ELISA method (Greiner Bio-One kit made in Germany), and to measure lipid profile (Triglycerides (TG; Intra- and inter-assay coefficients of variation of 4.5% and 5.3%), Total Cholesterol (TC; Intra- and inter-assay coefficients of variation of 6.5% and 3.2%), High-Density Lipoprotein (HDL; Intra- and inter-assay coefficients of variation of 3.3% and 4.8%) and Low-Density Lipoprotein (LDL; Intra- and inter-assay coefficients of variation of 3.6% and 6.8%)) the enzymatic method (Hitachi Kit, Tokyo, Japan) was considered based on the standard laboratory procedures. The direct competitive immunoassay method was used to assess Vit D levels.

### Statistical methods

The SPSS software (version 26) was used at a significance level of *P* < 0.05 to perform the statistical analyses. The mean and standard deviation of variables were measured using descriptive statistics, and the Shapiro–Wilk test was used to determine the normality of distribution. Within-group changes were examined using the dependent and independent t-tests and the difference in Differences (DID or DD) (Pre-2–Pre 1). The repeated-measures ANOVA and Bonferroni's post hoc tests were used for between-group comparisons.

## Results

Table 1 shows the anthropometric characteristics of the subjects. Table 1 shows that in the experimental, BW, and BMI were significantly lower; nevertheless, these variables increased in the control; yet, this difference was not statistically significant. Furthermore, when comparing the before to after alterations between experimental and control, the independent t-test revealed significant differences in BFP (*P* = 0.023) and WHR (*P* = 0.010). The results indicate that Vit D supplementation significantly reduced BW (*P* = 0.047), BMI (*P* = 0.044), BFP (*P* = 0.001), and WHR (*P* = 0.001) in the experimental group. Also, the results indicated that carbohydrate (*P* = 0.446; *P* = 0.689), protein (*P* = 0.563; *P* = 0.734), and lipid (*P* = 0.737; *P* = 0.492) were not significant in experimental and control, respectively. Additionally, the results of the independent t-test showed no significant difference in carbohydrate (*P* = 0.342), protein (*P* = 0.534), and lipid (*P* = 0.764) between experimental and control in the before and after.

**Table 1:** Mean ± SD of anthropometric indices before and after the intervention among the groups

Variables	Experimental group (n = 11)		Control group (n = 11)		*P* Value ^a^
	Before	After	Before	After	
Age (years)	26.90 ± 4.18	**–**	26.18 ± 4.42	**–**	
Height (cm)	165.45 ± 4.98	**–**	164.01 ± 6.01	**–**	
BW (kg)	76.68 ± 4.08	75.13 ± 4.87	74.12 ± 3.23	75.16 ± 3.27	0.788
*P* Value^b^	0.047*		0.051		
BMI (years)	28.03 ± 1.87	27.48 ± 2.07	27.63 ± 2.01	27.94 ± 1.90	0.419
*P* Value^b^	0.044*		0.064		
BFP (%)	29.60 ± 3.44	27.54 ± 2.60	28.60 ± 2.57	29.46 ± 2.96	0.023^¥^
*P* Value^b^	0.001*		0.066		
WHR (cm)	0.87 ± 0.022	0.85 ± 0.031	0.87 ± 0.045	0.88 ± 0.042	0.010^¥^
*P* Value^b^	0.001*		0.054		
CHO (gr/Day)	450.23 ± 23.12	446.21 ± 28.17	452.31 ± 30.07	453.14 ± 20.76	0.342
*P* Value^b^	0.446		0.689		
Protein intake (gr/Day)	120.14 ± 5.14	118.28 ± 6.23	122.52 ± 4.64	123.16 ± 7.61	0.534
*P* Value^b^	0.563		0.734		
Lipid intake (gr/Day)	96.23 ± 6.14	95.18 ± 3.17	95.14 ± 4.25	97.23 ± 4.04	0.764
*P* Value^b^	0.737		0.492		

*BW* Bodyweight, *BMI* body mass index, *BFP* body fat percentage, *WHR* waist–hip ratio, *CHO* carbohydrate

*P* values superscript with “a” is calculated using an independent t-test for comparing Δ between groups

*P* values superscript with “b” is calculated using dependent t-test for comparing pre-test and post-test within groups

*Significantly different comparing after and before

^¥^Significantly different between time points

Table 2 shows significant increases in lipid profile levels (except for HDL) in the experimental and control following the EEE (comparing Pre 1 and Post 1). Following the EEE, significantly higher serum lipid profile levels (expect for HDL) were reported in both the control and experimental groups after six weeks of Vit D supplementation (comparison of Pre 2 and Post 2). Also, in the experimental, the increase in TC, TG, and LDL in Post 2 was significantly lower compared to Post 1. Meanwhile, the increase in HDL levels in Post 2 was significantly higher than in Post 1. However, significantly lower TC (*P* = 0.001; *P* = 0.001), TG (*P* = 0.048; *P* = 0.001), and LDL (*P* = 0.001; *P* = 0.001); while significantly higher HDL (*P* = 0.001; *P* = 0.001) were observed in the experimental group compared to the control in both Pre 2 and Post 2; receptively.

**Table 2:** Lipid profile levels at different time points in experimental and control

Variables		Pre 1	Post 1	Pre 2	Post 2	*P* Value^a^
TC (mg/dl)	Experimental	227.12 ± 2.11^α£€^	242.81 ± 1.88^£€^	223.10 ± 1.73^€^	233.11 ± 2.19	0.001^¥^
	Control	226.72 ± 2.19^α€^	241.51 ± 1.99^£^	227.90 ± 1.86^€^	240.81 ± 2.27	0.001^¥^
*P* Value^b^		0.141	0.083	0.002*	0.001*	
TG (mg/dl)	Experimental	214.81 ± 1.77^α£€^	232.09 ± 1.10^£€^	208.09 ± 1.64^€^	223.18 ± 1.25	0.001^¥^
	Control	213.18 ± 1.77^α€^	231.27 ± 2.10^£€^	211.11 ± 1.67^€^	230.27 ± 2.10	0.001^¥^
*P* Value^b^		0.062	0.152	0.048*	0.001*	
LDL (mg/dl)	Experimental	204.06 ± 1.70^α£€^	225.17 ± 1.70^£€^	193.20 ± 1.57^€^	213.11 ± 1.87	0.001^¥^
	Control	203.27 ± 1.80^α€^	226.09 ± 1.86^£€^	205.27 ± 1.90^€^	230.21 ± 1.64	0.001^¥^
*P* Value^b^		0.139	0.204	0.001*	0.001*	
HDL (mg/dl)	Experimental	27.05 ± 1.67^α£€^	23.18 ± 1.77^£€^	37.14 ± 1.69^€^	33.21 ± 1.53	0.001^¥^
	Control	28.17 ± 1.83^α€^	23.27 ± 1.63^£€^	27.34 ± 1.42^€^	23.41 ± 1.62	0.001^¥^
*P* Value^b^		0.111	0.909	0.001*	0.001*	

*TG* Triglycerides, *TC* total cholesterol, *HDL* high-density lipoprotein, *LDL* low-density lipoprotein, *Vit D* Vitamin D

*P* values superscript with “a” is calculated using repeated measures ANOVA test for comparing different time points; *P* values superscript with “b” is calculated using independent t-test for comparing between groups at each time points

*Significantly different comparing Pre and Post

^¥^Significantly different between time points

^α^Significantly different compared with Post1 within the group

^£^Significantly different compared with Pre 2 within the group

^€^Significantly different compared with Post 2 within the group

**Fig. 3:**
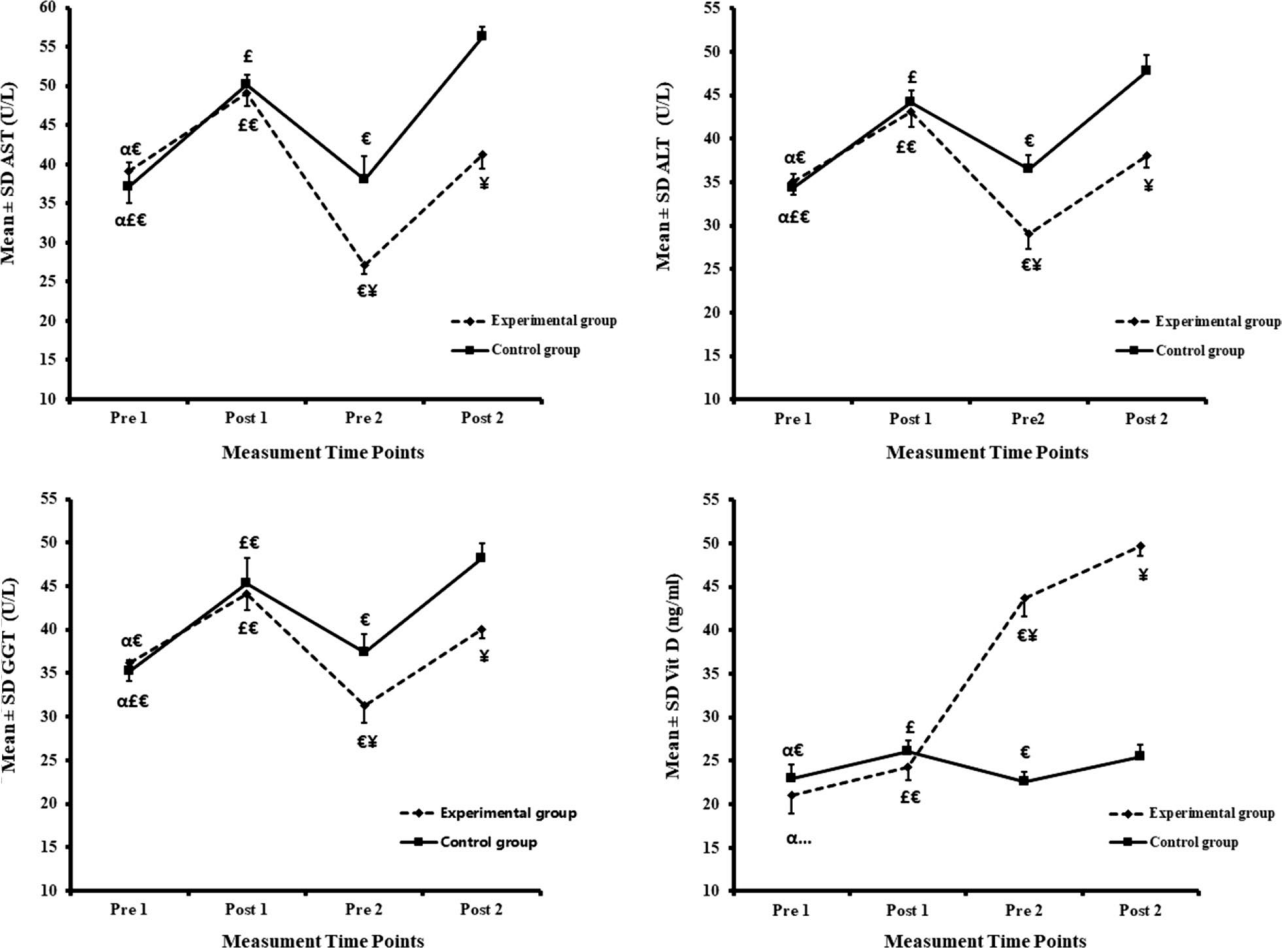
Liver enzymes and Vit D levels at different time points in experimental and control. *P* values superscript with “a” is calculated using Repeated measures ANOVA test for comparing different time points; *P* values superscript with “b” is calculated using independent t-test for comparing between groups at each time points. ^α^: Significantly different comparing with post1 within the group. ^£^: Significantly different comparing with pre 2 within the group. ^€^: Significantly different comparing with post 2 within the group. ¥: Significantly different comparing experimental and control

Figure 3 shows significant increases in liver enzymes (AST, ALT, and GGT) and Vit D levels in both experimental and control following the EEE (comparing Pre 1 and Post 1). Also, after six weeks of Vit D supplementation, serum levels of AST, ALT, GGT, and Vit D increased significantly in both experimental and control following EEE (the comparison of Pre 2 and Post 2). In experimental, however, the increase in AST, ALT, and GGT was much lower in Post 2 than in Post 1, and the increase in Vit D levels was significantly higher in Post 2 than in Post 1.

Compared with control, the results of the independent t-test showed significantly lower ALT (*P* = 0.001; *P* = 0.001), AST (*P* = 0.001; *P* = 0.001), and GGT (*P* = 0.001; *P* = 0.001); while significantly higher Vit D (*P* = 0.001, *P* = 0.001) in the experimental in both Pre 2 and Post 2; receptively (Fig. 3).

## Discussion

This study aimed to investigate the effect of a short-term Vit D supplementation on the alterations of liver enzymes (AST, ALT, and GGT) and lipid profile following EEE in overweight women with NAFLD. The results of our study indicate significantly reduced BW, BMI, BFP, and WHR in the experimental group following six-week Vitamin D supplementation. In parallel to our study, Hoseini et al. (2016) reported that high doses of vitamin D could significantly reduce BW, BMI, and visceral fat in rats with metabolic syndrome [17]. Vitamin D is a fat-soluble vitamin stored in larger adipose tissues after synthesizing and entering the bloodstream, releasing it at a slower rate. low levels of vitamin D might impair insulin function, glucose metabolism, and other metabolic processes in the adipose tissue, which might be another mechanism in the association of anthropometric changes following vitamin D supplementation [17, 18].

Interestingly, the results of this study showed significantly increased liver enzymes and lipid profile (except for HDL) following EEE. According to the results of other studies, vigorous exercise training increases mitochondrial oxygen consumption [19] and free radicals production, which leads to fat peroxidation [20], membrane-dependent enzyme dysfunction, and the destruction of the cell membrane [21]. Therefore, altered liver enzymes and lipid profiles could indicate the leakage of cell continents and structural cell damage [20]. Besides, eccentric contraction (e.g., running on a negative slope) exerts a greater force on the muscles, leading to muscular and hepatic cell damage and changed serum levels of liver enzymes [22, 23].

Also, the results of this study indicated that six-week Vit D supplementation significantly reduces the alteration of TC, TG, LDL, and HDL following EEE. Previous studies have investigated the effect of long-term vitamin D supplementation on the lipid profile in NAFLD patients [24, 25]. However, limited studies have investigated the effect of short-term Vit D supplementation on the EEE-induced alterations in NAFLD patients [26]. The possible mechanisms might involve the role of Vit D in increasing the lipoprotein lipase activity in adipose tissue [27]. Elevated Vit D levels might also be associated with inhibiting serum parathyroid hormone; In-vitro studies have shown that parathyroid hormone can reduce lipolysis [28]. Besides, Vit D regulates calcium homeostasis. Carmelite et al. (2015) showed that Vit D decreased hepatic triglyceride secretion probably via increasing calcium levels [29]. In general, Vit D reduces fatty acid absorption by making calcium-fatty acid soap [30]. Calcium also binds with the bile acids and causes fecal excretion, and the production of new bile acids lowers serum cholesterol levels. Therefore, Vit D might reduce cholesterol, triglyceride, and LDL levels by increasing calcium absorption [29, 31].

Additionally, the results of this study show the efficacy of vitamin D supplementation in reducing the EEE-induced liver enzyme alterations. Previous studies have examined the beneficial effects of long-term Vit D supplementation on liver enzymes in NAFLD patients [32, 33]. The primary protective mechanism of the effects of Vit D on reducing the liver enzymes following EEE still needs to be elucidated. However, insulin resistance might be among the reasons for the high liver enzymes [34]; insulin resistance activates the lipolysis and the flow of fatty acids to the liver via various factors, which may cause further liver damage [35]. Short-term vitamin D administration could improve insulin, glucose, and insulin resistance in NAFLD patients consequently improving the liver enzymes [36]. Also, Vit D prevents fat accumulation in the liver by inhibiting lipogenesis, enhancing fat oxidation, and regulating the circulation of free fatty acids [37].

In addition, a significant increase in Vit D level was observed following EEE. Since Vit D is a fat-soluble vitamin stored in adipose tissue and liver cells [33], and the cell membrane comprises two phospholipid layers, the higher Vit D levels after EEE might be higher linked to the cell membrane damage [26, 38].

### Strengths and limitations

This placebo-controlled, single-blinded, randomized study tried to find the answers to the novel questions. The study had a low dropout rate; however, the small sample size caused by COVID-19 and the lack of financial support that restricted us from measuring gene expression and other blood indicators are the limitations of this study. It is recommended to be considered in further research.

## Conclusion

Generally, short-term Vitamin D supplementation could regulate the perturbed liver enzymes and lipid profile induced by EEE. Therefore, Vit D supplementation could be recommended to NAFLD patients to control the alteration of liver enzymes and lipid profile following eccentric exhaustive activities.

## Data Availability

The datasets analyzed during the current study are not publicly available due to the guidelines of the Ethics Committee of the Kermanshah Razi University that proved this study regarding privacy/ethical restrictions but are available from the corresponding author on reasonable request.

## Abbreviations

EEE: Eccentric exhaustive exercise
NAFLD: Non-alcoholic fatty liver disease
BW: Bodyweight
BMI: Body mass index
BFP: Body fat percentage
WHR: Waist–hip ratio
Vit D: Vitamin D
AST: Aspartate aminotransferase
ALT: Alanine aminotransferase
GGT: Gamma-glutamyl transferase
TG: Triglycerides
TC: Total cholesterol
HDL: High-density lipoprotein
LDL: Low-density lipoprotein
